# Enhancing Tensile Modulus of Polyurethane-Based Shape Memory Polymers for Wound Closure Applications through the Addition of Palm Oil

**DOI:** 10.3390/polym16131941

**Published:** 2024-07-07

**Authors:** Sirasit Kampangsat, Todsapol Kajornprai, Warakarn Tangjatuporn, Nitinat Suppakarn, Tatiya Trongsatitkul

**Affiliations:** 1 School of Biomedical Innovation Engineering, Suranaree University of Technology, Nakhon Ratchasima 30000, Thailand; sirasit.kampangsat@hotmail.com; 2 School of Polymer Engineering, Institute of Engineering, Suranaree University of Technology, Nakhon Ratchasima 30000, Thailand; kajornprai.t@gmail.com (T.K.); nitinat@g.sut.ac.th (N.S.); 3 Research Center for Biocomposite Materials for Medical Industry and Agricultural and Food Industry, Suranaree University of Technology, Nakhon Ratchasima 30000, Thailand; 4 School of Surgery, Institute of Medicine, Suranaree University of Technology, Nakhon Ratchasima 30000, Thailand; warakarnt@sut.ac.th

**Keywords:** shape memory polymer, polyurethane, polycaprolactone, wound closure, palm oil

## Abstract

Thermo-responsive, biocompatible polyurethane (PU) with shape memory properties is highly desirable for biomedical applications. An innovative approach to producing wound closure strips using shape memory polymers (SMPs) is of significant interest. In this work, PU composed of polycaprolactone (PCL) and 1,4-butanediol (BDO) was synthesized using two-step polymerization. Palm oil (PO) was added to PU for enhancing the Young’s modulus of the PU beyond the set criterion of 130 MPa. It was found that PU had the ability to crystallize at room temperature and the segments of individual PCL and BDO polyurethanes crystallized separately. The crystalline domains and hard segment of PU greatly affected the tensile properties. The reduction of crystalline domains by the addition of PO and deformation at the higher melting temperature of the crystalline PCL polyurethane phase improved the shape fixity and shape recovery ratios. The new irreversible phase, raised from the permanent deformation upon stretching at the between melting temperature of the crystalline PCL and BDO polyurethanes of 70 °C, resulted in a decrease in shape fixity ratio after the first thermomechanical stretching–recovering cycles. The demonstration of PU as a wound closure strip showed its efficiency and potential until the surgical wound healed.

## 1. Introduction

SMPs are a type of smart polymer that has become a significant category of responsive materials for medical implants and biomedical and engineering applications [1,2,3,4]. The key feature of SMPs is their ability to be programmed into a temporary shape by deformation at the programming temperature (T_p_) and then return to their original shape when exposed to external stimuli such as heat, electricity, enzymes, etc. [4,5]. Thermo-responsive SMPs typically consist of two phases: a thermally reversible phase (e.g., hydrogen bonds and glassy-amorphous domains) that maintains a temporary shape and an irreversible phase (e.g., chemical/physical cross-links and crystalline domains) that enables the recovery of the original shape [4,6]. The reversible phase is responsible for fixing the temporary shape after sample deformation by cooling and can be reversed by heating across the shape-recovery temperature (T_r_) [4,7,8]. The permanent network or irreversible phase stores the elastic energy that drives the shape recovery upon stimulation and determines the original shape [4,6]. For polymer materials, the glass transition temperature (T_g_) of the amorphous segment or melting temperature (T_m_) of crystalline domains can be used as the T_r_ [4]. These properties allow SMPs to be used in many applications, including orthodontic wires [9], orthodontic aligners [7], smart plaster [10], smart splints [3], sutures [4], and wound closure strips [4].

Recent advancements in SMPs hold promise for use as wound closure strips. Wound closure is a critical step in wound healing, as bringing the tissues surrounding a surgical wound into close proximity facilitates efficient healing, reducing the risk of infection [11]. Currently, various methods, such as sutures or surgical staples, are employed for wound closure [12]. However, while these methods are effective, they may also result in increased pain and/or additional scarring [13]. This drawback has spurred research into alternative products, including adhesive-based solutions like glue wounds [14] or Steri-strip [15]. Nevertheless, these alternatives present challenges such as application difficulties and limitations in closing certain types of wounds, rendering them unable to address all scenarios [16,17].

For wound closure applications, the designed wound closure strip must be tough and chemically safe to avoid causing skin irritation. More importantly, the smart strip needs to effectively bring the skin together without losing its shape. In other words, from the material property point of view, the Young’s modulus of the strip must be greater than that of the skin. This modulus is required to ensure that the strip maintains its shape while pulling the two sides of the wound together. For wound closure applications, the smart strip needs to effectively bring the skin together without losing its shape, similar to that of a suture. Greenwald et al. [18] suggested that the mechanical properties of monofilament sutures (Maxon or PDS), which are currently popular for surgical wound closure, include a Young’s modulus of 130 MPa. This modulus is greater than that of human skin and is more than sufficient to hold surgical wounds together.

For the thermo-responsive SMP wound closure strip, the T_r_ is also important. Heat is applied on the strip to recover its primary shape. At T_r_, the strip shrinks, providing the force necessary to pull the skin around the wound together. Ideally, the T_r_ should be slightly above the physiological temperature. The risk of thermal injury for most mammalian tissues depends on both temperature and the duration of exposure. It was reported that tissue underwent irreversible damage after 60 min of exposure at 43 °C. The same level of damage occurred with 30 min of exposure at 44 °C or 15 min at 45 °C, indicating that the heating time halved for each degree increase in temperature [19]. Therefore, enhancing the modulus of PU-based SMPs to meet the requirements for wound closure with a low T_r_ close to human temperature, the body core temperature of 36–38 °C [19], is a crucial attribute to consider for achieving practical application.

PU is a promising candidate for meeting these requirements due to its adaptable mechanical properties, which can be tailored to fit specific applications [1,20,21]. In an early application [22], a PU-based SMP was employed for a custom-designed spoon handle intended for the physically handicapped. In this use case, the spoon handle was heated and shaped to match an individual hand shape, and then its shape was fixed at room temperature to provide a comfortable and personalized fit. PU is composed of linear soft and hard segments linking with urethane bonds, formed by the reaction of a diisocyanate hard segment with a polyol soft segment [23,24,25]. The properties of PU-based SMPs largely depend on the structure, shape, and crystallization of hard and soft segments. The symmetrical diisocyanate tends to form a crystallizable structure [25]. Ahmad, M. et al. [24] synthesized various PU-based SMPs by utilizing five different polyols as soft segments and two different diisocyanate structures (liner aromatic bent structure of 4,4′-diphenylmethane diisocyanate (MDI) and cycloaliphatic isophorone diisocyanate (IPDI)) as hard segments. A higher MDI content results in SMPUs with enhanced shape-memory properties. Because IPDI had short-range conformational motions and restricted segmental movements of soft-segment chains, increasing IPDI content led to a weaker shape-memory effect while the fixity rate increased. The T_r_ of the SMPUs can be adjusted between 30 and 45 °C by altering the MDI-to-IPDI molar ratio, making them suitable for medical device applications due to their proximity to body temperature. In the literature, it was found that manipulating the hard-segment contents in the PU-based SMPs played a crucial role in physical characteristics and their shape memory properties [6,26]. A high degree of crosslinking is essential for producing PU-based SMPs with good thermomechanical properties and shape-memory effects [24]. Kim, B.K. et al. [6] reported that, as a major component was the soft segment, the Young’s modulus and tensile strength decreased as the block lengths increased. In addition, the low content of the hard segment was detrimental to shape recovery. The BDO hard segment was used to synthesize PU-based SMPs with good mechanical and shape memory properties [6,26]. Lee, B.S. et al. [26] revealed that maximum stress, tensile modulus, and elongation of PU-based SMPs increased significantly as the BDO hard-segment content moved beyond 30 wt%. In addition, the high shape recovery of around 80–95% was achieved at 30–45 wt% of hard-segment contents.

PCL is a soft semi-crystalline polymer with biodegradable and biocompatible properties, having low T_g_ and T_m_ at around −60 and 60 °C [27], respectively. Thus, this material exhibits flexible and rubber-like characteristics at room temperature, and it is applicable for thermal stimulation of shape recovery close to human temperature. PU-based SMPs composed of PCL soft segments and BDO hard segments demonstrated a shape recovery of over 94%, with a wide T_r_ ranging from 40 to 65 °C. However, the shape fixity is highly dependent on both the hard-segment content and the temperature programming [28]. Their crystallinities, mechanical properties, and shape memory properties varied depending on the molecular weight of PCL and the molar ratio of soft and hard segments [28,29].

It is well known that PU comprises block copolymers composed of polyester or polyether segments connected by urethane linkages (–NHCOO–), which are formed by the reaction between isocyanate (–N=C=O) and hydroxyl (–OH) groups [30]. Several research studies have explored the use of palm oil (PO) for PU preparation [20,31,32]. The addition of PO facilitates the promotion of urethane bond formation [31], influencing the creation of hard segments, which increases both the Young’s modulus and elongation as well as achieves good shape fixity and shape recovery ratios [20,32]. However, PO primarily consists of triglycerides (Tri-Gs), making it necessary to introduce hydroxyl groups to the Tri-G molecule for urethane reactions [33]. PO-based polyols can be synthesized using different methods [30,34,35,36,37]. Examples of palm oil applications in the synthesis of polyols for the preparation of rigid PU can be found in the literature [30,32,34,35,36]. Such polyols are usually obtained by transesterification using various agents, such as diethanolamine (DEA) [38,39] and dibutyltin dilaurate (DBTDL) [20,40].

This work aimed to synthesize PU-based SMPs composed of PCL soft segments and BDO hard segments. The Young’s modulus of 130 MPa—derived from the linear elastic mechanical properties of synthetic suture wires [18,41]—was selected for the criterion of the thermo-responsive wound closure strip in this study. The effect of PO hard-segment contents on the thermal, mechanical, and shape memory properties of PU was characterized, and the proof of concept for the use of PU-based SMPs in thermo-responsive wound closure strip application was demonstrated. Our findings suggest that PU-based SMPs with the addition of PO hold significant promise for improving wound closure techniques, potentially leading to better clinical outcomes.

## 2. Materials and Methods

### 2.1. Materials

The following chemicals were acquired from Sigma-Aldrich (St. Louis, MO, USA): PCL with an average molecular weight (M_n_) of 2000 g/mol, 4,4′-methylenebis (cyclohexyl isocyanate) (HMDI) with a purity of 90% and a variety of isomers, BDO with a purity of 99%, and DBTDL with a purity of 95%. PO, an edible palm oil commercially available in the Thailand market, was a product from Lam Soon Public Co., Ltd. (Bangkok, Thailand), with a specific gravity of 0.9202 g/cm^3^ [37]. All the chemicals were used as received. In addition, an artificial 3-layer human skin model (model number SP01) was purchased from Shawn Science Manufacturing (Atlanta, GA, USA). 

### 2.2. Synthesis of PU and Sample Preparation

PU was synthesized via a standard two-step pre-polymer method in the absence of organic solvent. The chemical reaction for the synthesis of PU is described elsewhere [42,43,44,45,46]. In the first step, the pre-polymer was prepared by adding the PCL as a soft-segment precursor and HMDI as a coupling agent in a round-bottom flask, heating to the temperature of 110 °C in a pre-heated silicone oil bath, and stirring using a magnetic stirrer at a speed of 190 rpm for 2.5 h. After that, PO as a hard segment was introduced into the mixture and mixed for a further 2 h. The reaction was carried out under the vacuum condition to remove the H_2_O molecules as a by-product. In the second step, the pre-polymer was then reacted with BDO as a hard segment and one drop of DBTDL as the catalyst for a further 20 min. The molar ratio of HMDI and BDO was fixed, whereas the molar ratio of PCL and PO was varied. Table 1 summarizes the molar ratio of all reagents and the sample code name and hard segment content (HSC). The HSC was calculated from the following equation [28]:

(1) $$HSC(\%)=\frac{\left(n_{PO}\times M_{PO})+{(n}_{HMDI}\times M_{HMDI})+{(n}_{BDO}\times M_{BDO}\right)}{{(n}_{PCL}\times M_{PCL})+{(n}_{PO}\times M_{PO})+{(n}_{HMDI}\times M_{HMDI})+(n_{BDO}\times M_{BDO})}\times 100$$

where $M_{PO}$, $M_{HMDI}$, $M_{BDO}$, and $M_{PCL}$ are the molecular weights of each component and $n_{PO}$, $n_{HMDI}$, $n_{BDO},$ and $n_{PCL}$ are the mole numbers of the components in the PU molecule.

For the mechanical, thermal, and shape memory tests, the resulting PU was hot-pressed into dumbbell-shaped specimens with a thickness of 0.5 mm, according to ASTM D638-14 [47], in a compression molding (LabTech Model LP20-B, Samutprakarn, Thailand) at 150 °C under the pressure of 15 MPa for 5 min. The rectangular-shaped strip with a dimension of 5 × 20 mm was cut from the narrow section of a dumbbell-shaped specimen to demonstrate the wound closure strip application. All samples were kept at room temperature before characterization.

### 2.3. Characterizations

The Fourier-transform infrared spectroscopy (FT-IR) (BRUKER, VERTEX 70, Billerica, MA, USA) analysis was performed in a transmission mode to verify the success of the PU synthesis. The FT-IR spectra of the PU specimen were recorded with a resolution of 4 cm^−1^ and scan number of 64 in the range of 4000 to 500 cm^−1^ for each measurement. The thermal behaviours of PU were determined using Differential Scanning Calorimetry (DSC) (METTLER TOLEDO, DSC3, Columbus, OH, USA). The sample of around 5 mg was cut and sealed in an aluminium pan. The sample was equilibrated at −100 °C for 2 min before heating to 120 °C with the heating rate of 5 °C/min under 50 mL/min N_2_ flow. The T_g_, T_m_, and melting enthalpy ($∆H_{m}$) of PU were acquired from the first DSC heating scan to relate to the mechanical properties and shape memory characteristic of PU. The crystallinity percentage of the PCL phase in PU was calculated by the following Equation [48]:

(2) $$X_{c}\left(\%\right)=\frac{∆H_{m}}{w\times ∆H_{m}^{{}^{\circ}}}$$

where the w is the weight fraction of the PCL phase in the PU, and $∆H_{m}^{{}^{\circ}}$ is the 100% melting enthalpy of PCL crystals, having a value of 140 J/g [49].

The X-ray diffraction (XRD) (Bruker, D8 Advance, Billerica, MA, USA) measurement was conducted at room temperature using a Cu-Kα X-ray source. The diffraction pattern was recorded over a 2*θ* range from 10° to 80°. Thermogravimetric analysis (TGA) (Mettler Toledo, TGA/DSC1, Columbus, OH, USA) was employed to assess the thermal stability and decomposition temperature of the PU. The samples were heated from 35 to 500 °C with a heating rate of 10 °C/min under a N_2_ flow rate of 100 mL/min. The tensile properties were evaluated using a universal testing machine (UTM) (Instron 5565, Northwood, MA, USA) at a crosshead speed of 50 mm/min with a 5 kN load cell according to ASTM D638 [47]. The average value from 5 samples was reported.

**Figure 1:**
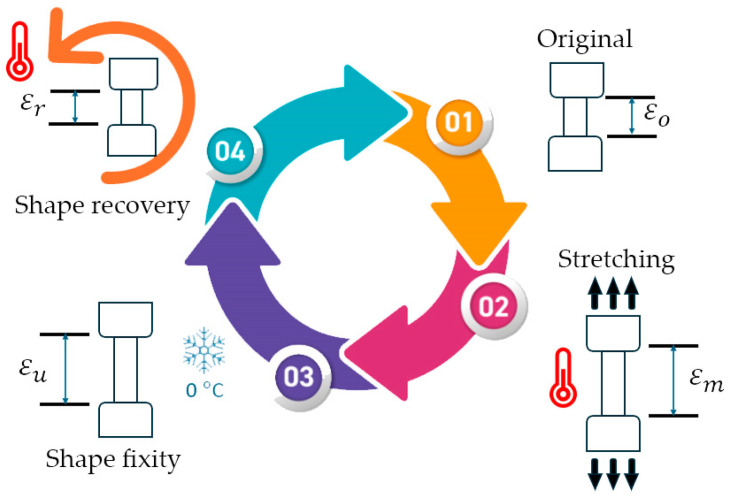
The thermomechanical program for the shape memory test of PU.

To evaluate the shape memory characteristics of the PU, the shape deformation was analyzed using the UTM coupled with a temperature-controlled chamber (Instron, 3119-406, Norwood, MA, USA). As shown in Figure 1, a dumbbell-shaped specimen was measured with the original length as the primary shape (step 1), denoted as $\varepsilon_{0}$. Then, the specimen was equilibrated at various T_p_ ranging from 40 to 80 °C inside the temperature-controlled chamber for 3 min before stretching to a specified length of 10 mm at a rate of 50 mm/min to form a temporary shape (step 2), denoted as $\varepsilon_{m}$. Subsequently, the specimen was rapidly cooled using cool pads for 1 min while the force was being maintained. Next, the specimen was removed from the clamps, conditioned in a cooler box (~0 °C) for 12 h, and the final length was measured (step 3), denoted as ($\varepsilon_{u}$). After that, the specimen was immersed in warm water with the corresponding T_r_ for 1 min. The changes in length of the specimen were measured, denoted as ($\varepsilon_{r}$). The shape fixity ratio ($R_{f}$) and the shape recovery ratio ($R_{r}$) were calculated using the following equation:

(3) $$R_{f}\left(\%\right)=\frac{\varepsilon_{u}}{\varepsilon_{m}}\times 100$$

(4) $$R_{r}\left(\%\right)=\frac{\varepsilon_{o}-\varepsilon_{r}}{\varepsilon_{o}}\times 100$$

where $\varepsilon_{o}$, $\varepsilon_{m}$, $\varepsilon_{u}$, and $\varepsilon_{r}$ are the stain length of the material at the initial length, after stretching to 10 mm at T_p_, after cooling for 12 h, and after recovery [8], respectively. At least 5 replications were averaged and reported.

**Figure 2:**
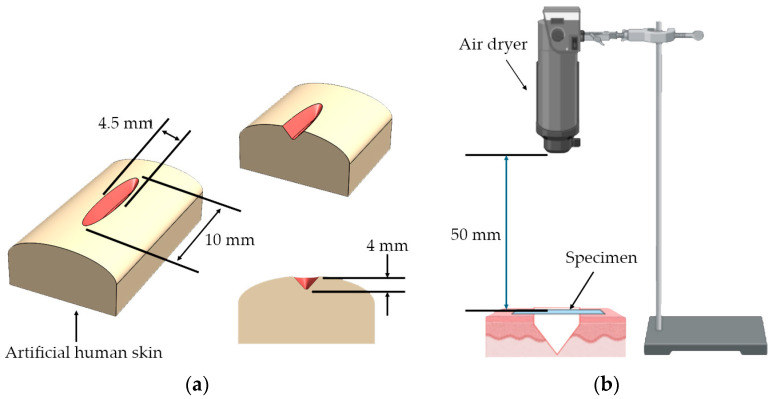
(**a**) Wound cutting dimension on artificial human skin and (**b**) the setup for the shape recovery of wound closure strip made from PU.

The proof-of-concept experiment for the smart wound closure strip performance on artificial human skin was designed based on information acquired from the finite element (FE) model from the literature [50,51,52,53]. A small open wound with a dimension of 10 mm in length and 4 mm in depth was created, as shown in Figure 2a. In order to demonstrate the ability of the PU wound closure strip to retain its shape when subjected to pulling forces from artificial human skin after undergoing shape recovery, PU-based shape memory polymers (SMPs) with a Young’s modulus of 130 MPa and above were utilized. A pre-stretched specimen at 70 °C with 10 mm was attached to the artificial human skin across the middle of the wound opening area. It was then heated with a hot-air dryer (HRHD01BK, HAN RIVER, Shenzhen Yunuo Supply Chain Co., Ltd., Shenzhen, China), which was set to heating level 3 for 30 s. The experimental setup is shown in Figure 2b. The air temperature monitored using a thermocouple at the surface of the artificial human skin was around 47 ± 0.3 °C. After recovery, it was kept in a room with a controlled temperature of 25 °C for 15 days to simulate the duration required for the wound healing process. The performance of the wound closure strip was assessed by capturing a sequence of photos both during and after the recovery process, and the distance between the wound edges was measured using the ImageJ program. The result was documented as the width of the wound opening at various intervals of observation. A minimum of 5 replications were taken, and an average value was reported.

## 3. Results and Discussion

### 3.1. FT-IR Analysis

**Figure 3:**
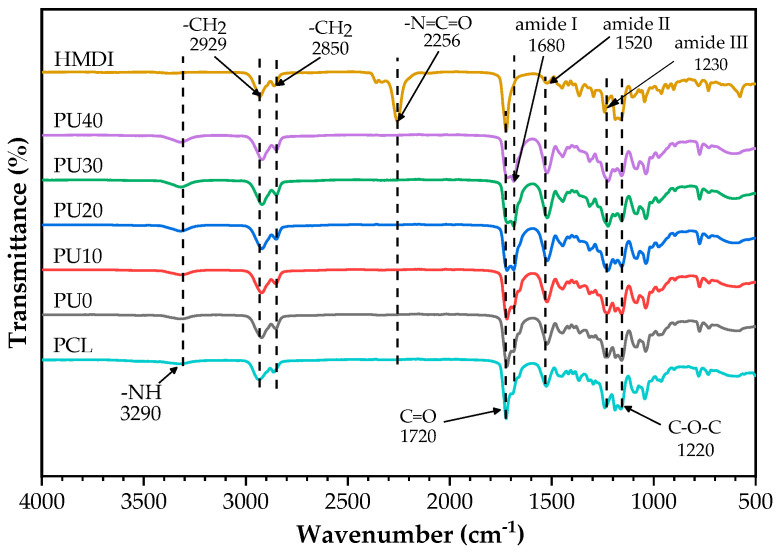
FTIR spectra of PCL, HMDI, and PU.

FTIR spectroscopy was employed to identify the functional groups present in PCL, HMDI, and PU molecules. Figure 3 illustrates their FT-IR spectra. In accordance with the literature, the spectrum of PCL demonstrates distinct absorption peaks at the wavenumbers of 1720 and 1220 cm^−1^, which correspond to the stretching of C=O and C–O–C bonds, respectively [45,54,55]. Additionally, the vibrations of –CH_2_ groups were seen as symmetric stretching at 2929 cm^−1^ and asymmetric stretching at 2850 cm^−1^ [55]. The FT-IR spectra of PU with various amounts of added PO revealed the characteristic absorption peaks of PCL, as previously mentioned, along with the identification of a newly emerging amine functional group. The peak detected at 3290 cm^−1^ corresponds to the stretching vibration of the –NH group in the urethane linkage. The peaks seen at 1680, 1520, and 1230 cm^−1^ can be ascribed to the amide I, amide II, and amide III vibrations, respectively, as documented in references [54,55,56]. There were no absorption peaks at 2256 cm^−1^ in any of the FT-IR spectra of PU, indicating the absence of the carbamate group (–N=C=O) of HMDI. Additionally, it was noted that the intensity of the peak at 1680 cm^−1^ corresponding to the amide I was significantly associated with the existence of chemical bonds formed between the isocyanate group and PO. The intensity of amide I at 1680 cm^−1^ tended to increase over the peak intensity of the C=O bond at 1720 cm^−1^ when the PO content increased, as clearly seen for PU20 to PU40. Comparable FT-IR spectra of PCL-based PU have been documented in various research studies [43,45,54,55].

### 3.2. DSC Analysis

**Figure 4:**
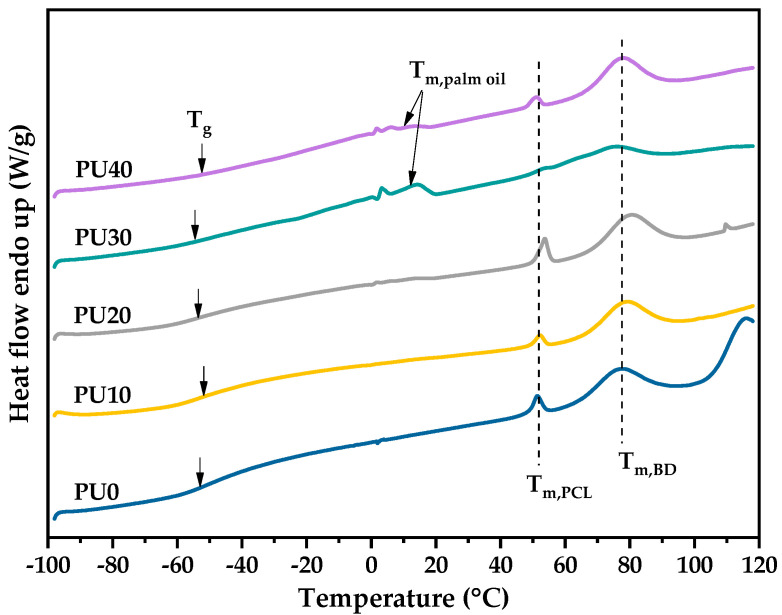
The 1st DSC heating thermogram of PU.

Figure 4 shows the first DSC heating scan of PU with various PO contents; all DSC parameters are summarized in Table 2. Of course, the crystallization of all PU samples was completed during the sample preparation process due to the absence of the cold-crystallization behavior upon the DSC heating scan. The lower T_m_ at around 50–54 °C was attributed to the melting of the crystalline polycaprolactone-polyurethane (PCL-PU) phase, and the higher T_m_ at about 74–80 °C belonged to the crystalline BDO polyurethane (BDO-PU) phase. The literature reported that the T_ms_ of PCL-PU was in the range of 40–60 °C [24,28,29]. Ping, P. et al. [29] revealed that the PCL-PU with the M_n_ of the PCL precursor lower than 2000 g/mol was non-crystalline and absent of the melting peak upon DSC heating scan. With the M_n_ of PCL above 2000 g/mol, PCL-PU could crystallize and exhibit a T_m_ of around 53.3 °C. These results are in agreement with the findings reported in this work, where the M_n_ of PCL was 2000 g/mol and the T_m_ of the PCL-PU phase was around 53 °C. In addition, the literature reported that the melting of BDO-PU occurred at high T_m_ [26]. Thus, it was confirmed that the lower and higher T_m_ corresponded to the melting of crystalline PCL-PU and crystalline BDO-PU phases, respectively. Moreover, the two melting peaks were observed for all PU samples, indicating that the PCL-PU phase and BDO-PU phase crystallized separately due to the different structural regularities and thermodynamic incompatibility.

Furthermore, a broad and small melting endotherm at the temperature range from 4 to 15 °C was attributed to the melting of PO [57,58] which was prominently observed at the higher PO content. The T_m_ of PO was not seen in PU10 due to the low PO content. Initial signs of PO melting began to appear in PU20, albeit with minimal deviations from the baseline. The distinct peak associated with PO melting became clearly observable starting from PU30 and above. This characteristic peak is indicative of the presence and influence of palm oil in the formulation. The addition of PO had insignificant effects on the T_g_s of PU due to it being far below room temperature. However, it was found to influence the crystallization ability and degree of crystallinity determined from the $∆H_{m}$ of both PCL-PU and BDO-PU phases. The %X_c_ of the PCL-PU phase was negligible (less than 1%) due to it being a minor phase (Table 1). As seen in Table 2, the $∆H_{m}$ of the BDO-PU phase for PU10 decreased as compared to the PU0 sample without the addition of PO, indicating the interruption of crystallization from PO. With increasing of the PO content, the clear T_m_ of PO indicated the phase separation. Thus, the crystallization ability of PU was enhanced, resulting in the increase of the $∆H_{m}$ of the BDO-PU phase. However, PU30 exhibited small $∆H_{m}$ for both PCL-PU and BDO-PU phases, showing a lower crystallinity of PCL-PU and BDO-PU phases.

### 3.3. XRD Analysis

**Figure 5:**
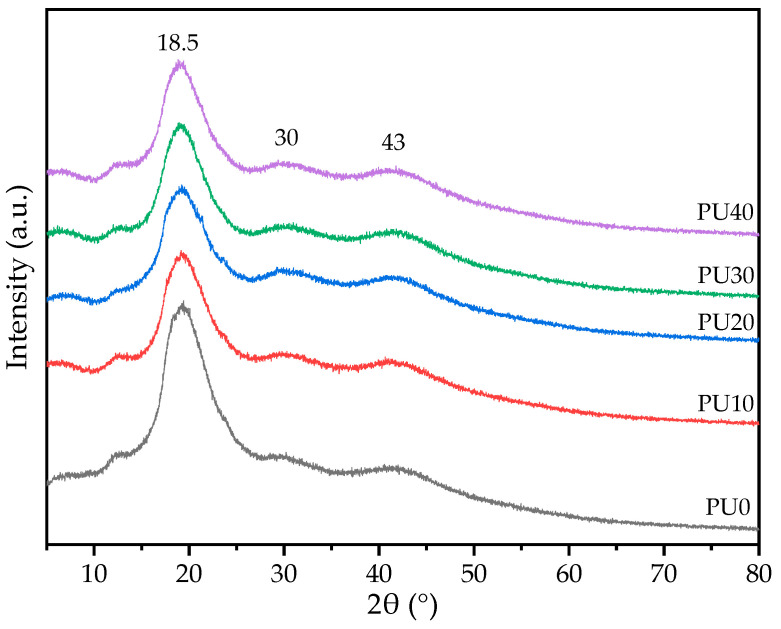
The XRD patterns showing the intensity of the crystalline diffraction peaks of PU with different PO contents.

XRD analysis was used to evaluate the crystalline state of PU samples. As shown in Figure 5, all PU samples presented a broad crystalline peak with an amorphous diffraction pattern in the XRD curves, indicating the crystalline structure developed during the sample preparation process. The crystalline diffraction peaks at 18.5°, 30°, and 43° were attributed to the characteristic pattern of the crystalline BDO-PU phase. A similar XRD diffraction pattern was found for the palm kernel oil-based PU [20]. Normally, PCL as a semi-crystalline polymer exhibits a sharp distinct diffraction peak at 21.6° and a small-intensity sharp diffraction peak at 24.1°, corresponding to the (110) and (200) lattice planes of the PCL [54,59], respectively. As the PCL precursor formed PCL-PU, the intensities of both crystalline peaks became broadened and decreased in magnitude [48]. This was because the hard segments in PU have higher polarity from isocyanate groups, short chain length, and molecular asymmetry, unlike the soft segments [48,60]. Consequently, the asymmetrical hard segment was difficult to crystallize and exhibited a lower crystallinity. However, in this case, BDO was a symmetric molecule and capable of forming a crystalline structure. Therefore, a broad diffraction peak crystalline PCL-PU phase could be overlapped with a broad diffraction pattern of the crystalline BDO-PU phase. A similar diffraction pattern of PCL and BO-based PU was observed by others [20,54]. The diffraction intensity of the crystalline PCL-PU phase reduced as the PO content increased due to the portion of PCL soft segments being reduced. This observation was inconsistent with the increase in peak intensity of the amide I group at 1680 cm^−1^ from the FTIR analysis (Figure 3). The PO phase could not crystallize because the samples were kept and measured at room temperature above the T_m_ of PO (4–15 °C) thereby resulting in a liquid phase and acting as a chain extender. The intensity of crystalline peaks of the BD-PU phase seemed to experience a negligible effect from the PO. Thus, there was a co-existence of the crystalline and amorphous phases in the PU samples. In addition, all peak positions of all crystalline diffraction patterns were located at the same 2θ angle. This evidence indicated that the crystal structure of the crystalline PCL-PU and BDO-PU did not change and confirmed the DSC results that both phases crystallized separately and that PO was not crystalline.

### 3.4. Tensile Behaviors

The tensile results of PU are summarized in Table 3. As the T_g_ of PU was far below room temperature, the PU samples were in a rubbery state. Without the addition of PO, PU0 exhibited ductile behavior, having a low Young’s modulus of 88.6 ± 3.20 × 10^−2^ MPa and low tensile strength of 10.15 ± 2.09 MPa, with the elongation at break of nearly 114%. By adding a small amount of PO, PU10 showed a lower Young’s modulus than that of PU0, while the elongation was maintained. With increasing the PO contents, the Young’s modulus increased and showed the maximum at 327.60 ± 2.80 × 10^−2^ MPa for the PU30 sample. Further increase of the PO content (PU40) resulted in a slight decrease of the Young’s modulus. However, the tensile strength and elongation were improved by the addition of PO.

The literature reported that the symmetrically hard segment could form the crystalline structure and affect the tensile properties [23]. In this case, the PCL-PU soft segment had a very low degree of crystallinity (~1%), and the coupling agent HMDI hard segment was asymmetric. Thus, the tensile properties of PU were influenced by the crystallinity of the BDO-PU phase and the HSC in the PU molecular chains (Table 1). The addition of PO had two contrasting effects on the tensile properties. DSC and XRD measurements indicated that the crystallinity of the BDO-PU phase decreases with the addition of PO, leading to a reduction in Young’s modulus and tensile strength for the PU10 sample. Although the crystallinity reduced, the Young’s modulus and tensile strength appeared to increase and reached their maximum in the PU30 sample. This outcome was due to the increase in HSC from the higher PO content through the filler-like effect, which played an important role in enhancing the mechanical properties of the PU, resulting in a stiffer material [23,46]. On the other hand, the reduction in crystallinity after the incorporation of PO improved the elongation of PU.

### 3.5. Shape Memory Properties

The PU-based SMPs underwent testing for their shape memory properties through two processes. The first process involved assessing the temperature effect to ascertain the effective operating temperature for shape memory. Additionally, the shape recovery of PU was tested to establish a temperature suitable for further assessing the functionality of the wound closure strip. In the second process, a repeatable cycle testing procedure was employed to identify the point at which the PU-based SMPs achieved a stable state. This stable state was determined to be the most suitable point for simulating wound closure strip testing.

#### 3.5.1. Effect of Temperature on Shape Memory Properties of PU

At room temperature, a crystalline portion was observed in PU as confirmed by DSC and XRD measurements. The crystalline domains of PCL-PU and BDO-PU as well as physical crosslinks or entangled chains served as an irreversible phase and controlled the shape memory effects in this situation. On the other hand, the amorphous PCL-PU phase, amorphous BDO-PU phase, PO, and un-crystallizable HMDI phase acted as a reversible phase upon the deformation and recovery processes. Five deformation temperatures of 40, 50, 60, 70, and 80 °C were chosen from the lower T_m_ of the PCL-PU phase, peak maximum T_m_ of the PCL-PU phase, greater T_m_ of PCL-PU phase, broad-based melting endotherm of the BDO-PU phase, and peak maximum T_m_ of the BDO-PU phase (Figure 4), respectively.

**Figure 6:**
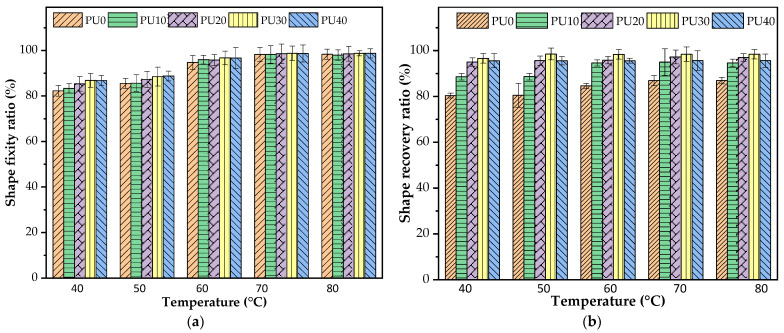
(**a**) Effect of temperature on shape fixity ratio of PU and (**b**) the recovery ability of PU at the corresponding T_p_.

Figure 6 shows the effect of T_p_ on the shape fixity and shape recovery ratios of PU at the corresponding T_p_. At the temperature lower than the T_m_ of the PCL-PU phase (T_p_ = 40 °C), the shape fixity ratio of PU0 without the addition of PO around 82% was lower than that of the PU with the addition of PO, as shown in Figure 6a. A similar result was observed at the T_p_ of 50 °C, but the shape fixity ratio of PU0 slightly improved to 86%. Compared to the T_p_ above the T_m_ of the PCL-PU phase (T_p_ = 60–80 °C), all PU samples showed a high shape fixity ratio, maintaining a value of around 95–98%, with minimal variation observed between samples with and without the addition of PO. Herein, at a temperature lower than the T_m_ of the PCL-PU phase (T_p_ = 40 °C), both crystalline PCL-PU and BOD-PU domains existed. In combination with the low T_p_, once the PU sample deformed, the high elastic energy stored in the crystalline domain forced the temporary shape to restore its original shape and resulted in a low shape fixity ratio. The overall crystalline domains of PU were reduced by the addition of PO. Thus, the shape fixity improved. In a similar manner, at the T_p_ above the T_m_ of the PCL-PU phase (T_p_ > 50 °C), the elastic energy storage was derived from what the crystalline PCL-PU domains had lost, making the polymer chains more flexible and mobile. Consequently, the temporary shape could be effectively maintained, resulting in a high shape fixity ratio.

As seen in Figure 6b, the shape recovery ratio for all PU samples appeared to be independent of T_p_. The addition of PO had a more significant effect on the shape recovery ability of PU. Regarding the T_p_ and T_r_ below the T_m_ of the PCL-PU phase (T_p_ = 40 °C), the existing crystalline domains of both PCL-PU and BDO-PU phases could be permanently deformed, and the irreversible deformation took place upon stretching, resulting in the low shape recovery in the PU0 sample. As the crystalline domains decreased with the addition of PO, the shape recovery of PU increased. Furthermore, when the crystalline domains of the PCL-PU phase melted (T_p_ > 50 °C), the increase in mobility allowed the material to be deformed more easily. The stress could then be transferred to the hard segment and stored. Clearly, with higher PO content, the shape recovery ratio increased due to the rise in HSC.

From the above results, both the crystalline domain and HSC had influences on the shape memory properties of the PU. The high crystalline domains showed a negative effect on both shape fixity and shape recovery. By decreasing the overall crystalline domain by stretching at the temperature above the T_m_ of the PCL-PU phase, the hard segment played the dominant role in enhancing the shape memory properties. Within the T_p_ range of 60–80 °C, the shape fixity ratio was maximized and still retained the temporary shape effectively, with a desirable shape recovery ability. The T_p_ and T_r_ at 70 °C were selected for a study of the repeatability of PU.

#### 3.5.2. Repeatability Study of PU-Based SMPs

The repeatability of PU-based SMPs was evaluated to determine the stability of shape memory parameters over multiple cycles and to ensure consistent functional behavior after the initial cycles. Many SMPs exhibited an improvement in shape recovery after the first deformation cycle [61,62,63]. This effect is well known as the “training effect” [64] and “cyclic hardening” [65].

**Figure 7:**
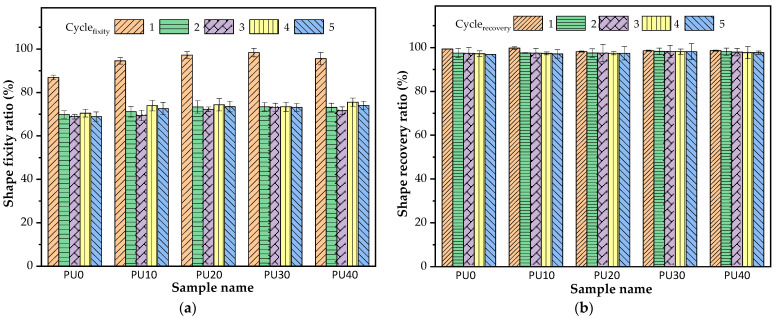
(**a**) Shape fixity ratio and (**b**) shape recovery ratio of PU at T_p_ and T_r_ of 70 °C under 5 thermomechanical stretching–recovering cycles.

However, as seen in Figure 7, the shape fixity ratio of all PU samples decreased and then remained stable after the first cycle of the repeatability test, while the shape recovery remained almost constant along with the five thermomechanical stretching-recovering cycles. In this case, the T_p_ of 70 °C was in the broad-based melting endotherm region of the BDO-PU phase. A number of crystalline domains of the BDO-PU phase existed in the PU sample. As aforementioned, the deformation that arose from the crystalline domains could cause permanent deformation upon stretching and consequently contributed to forming a new irreversible phase structure in the first deformation cycle, resulting in a greater HSC and higher elastic energy storage. Consequently, the shape fixity ratio was reduced in a second deformation cycle. The stable shape fixity ratio of the second to fifth deformation cycle indicated no occurrence of a new irreversible phase. Thus, the shape fixity ratio after the involuntary generation of greater HSC was around 69–75%. A similar observation was revealed in other PU-based SMP systems [66].

### 3.6. Proof-of-Concept of Thermo-Responsive Wound Closure Strip Application

**Figure 8:**
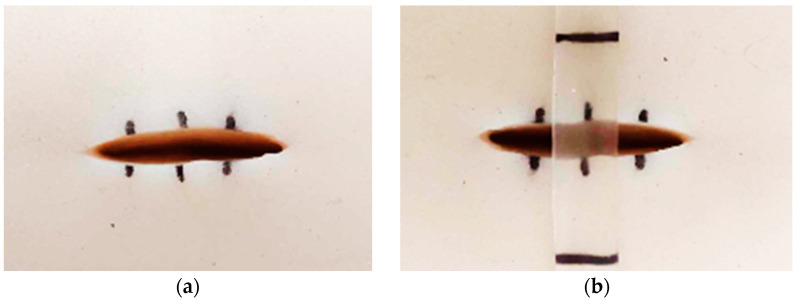
Digital image of (**a**) wound dehiscence on artificial human skin and (**b**) wound dehiscence with the wound closure strip made from PU.

**Figure 9:**
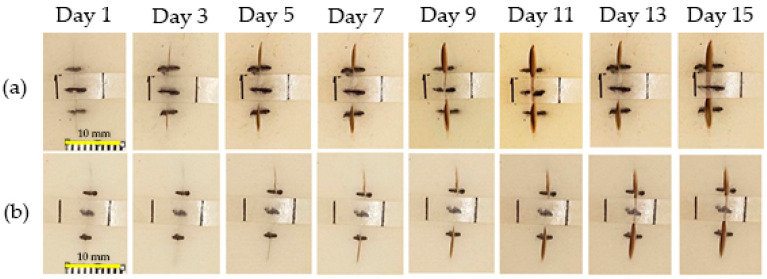
Digital image presentation of a wound closure strip made from (**a**) PU20 and (**b**) PU30 for maintaining the closure of wound dehiscence.

**Figure 10:**
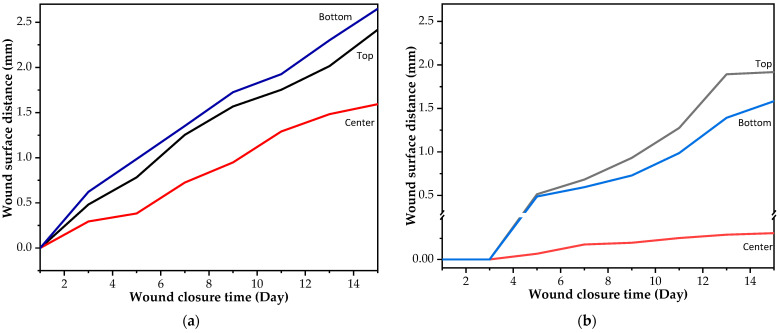
(**a**) Wound closure strip made of PU20 and (**b**) wound closure strip made of PU30.

As aforementioned, the criterion of Young’s modulus in this work was set as 130 MPa. From tensile results, PU20, PU30, and PU40 had Young’s modulus values of 155.60 ± 6.22 × 10^−2^, 327.60 ± 2.80 × 10^−2^, and 235.60 ± 0.82 × 10^−2^ MPa, respectively. The PU20 and PU30 samples were selected to demonstrate a thermo-responsive wound closure application due to the lowest value occurring at higher the criterion and the maximum value achieved among the PU samples, respectively. Figure 8 illustrates the wound dehiscence on artificial human skin, where the wound typically displays separation on either side. The specimen was positioned in the center of the wound on the artificial human skin. It was secured with pins at both ends to ensure it adhered to the artificial skin. Employing the principle of shape memory polymer recovery, the wound closure strip pulled the separated wound edges together, promoting closure. Figure 9 demonstrates the capability of using PU-based SMPs as wound closure strips. Both sides of the wound were effectively brought together for healing following the recovery of the PU-based SMPs to their primary shape. It was observed that the wound edges separated from each other after a few days due to the tension from the artificial human skin. The distance between the two sides of the separated wound edge is shown in Figure 10. The results indicated that, using PU20, the center distance of the wound edges began to separate on the first day and tended to increase over time, resulting in the appearance of a channel at the top and bottom parts of the wound dehiscence. Conversely, PU30, which has the highest Young’s modulus, displayed a small wound channel after 5 days, slightly increasing to 0.03 mm by day 15. The top and bottom parts, which were not attached to the wound closure strip, began to separate after 3 days. The results demonstrated that PU30 with a high Young’s modulus had a greater efficiency in maintaining wound closure than that of PU20. This suggested that thermo-responsive PU-based SMPs have the potential to be utilized to cover wounds until the surgical wounds heal.

From our point of view, a single wound closure strip made of PU-based SMPs with a Young’s modulus slightly above 130 MPa was not effective during the wound healing process. PU-based SMPs with a higher Young’s modulus had a significant impact on resisting the pulling force. Future research will focus more on balancing the Young’s modulus, mechanical properties, and shape memory properties of PU. The aspects of bio-adhesion between the PU surface and the artificial skin, as well as the sterilization process, were not addressed here. In the future, it will be essential to use a sterilization technique such as hydrogen peroxide, rather than relying on temperature, to maintain the temporary shape of the PU wound closure strip. Additionally, using bio-adhesives instead of pins in practical applications would be a favorable choice due to their improved ability to adhere to the skin and their non-destructive effect on live cells.

## 4. Conclusions

In conclusion, the aim of enhancing the tensile modulus of PU-based SMPs was achieved by adjusting the ratio of PO to PCL from 0 to 40 molar ratio, targeting a Young’s modulus of 130 MPa for wound closure applications. The successful incorporation of palm oil into the PU-based SMP structure was confirmed by FTIR analysis. Mechanical testing demonstrated that the target modulus was achieved by incorporating PO, starting from PU20. A significant increase in Young’s modulus was observed, from 88.60 ± 3.20 × 10^2^ MPa at PU0 to 327.60 ± 2.80 × 10^2^ MPa at PU30, indicating a nearly 2.7-fold increase due to PO, attributed to its filler-like effect and increased hard-segment content.

Furthermore, the shape fixity and recovery properties of PU-based samples were found to be influenced by the Tp and hard-segment content, both of which increased with PO content. In the proof-of-concept experiment, effective wound closure without gaps on the first day was demonstrated by PU20 and PU30 wound closure strips. By day 15, PU20 exhibited a 1.5 mm increase in wound size, whereas PU30 showed only a 0.03 mm increase, highlighting PU30’s approximately 50-fold superior wound-holding ability over PU20.

While promising results were achieved in this study, challenges remain. The relatively high recovery temperature of the samples may require careful adjustment to optimize practical applications alongside mechanical properties. Furthermore, investigation into the cytotoxicity of PO-modified PU-based SMPs is crucial for ensuring their safety and suitability in medical applications. Addressing these challenges will be pivotal for advancing smart wound closure strips based on PO-modified PU-based SMPs.

## Figures and Tables

**Table 1:** The formulation, sample code name, and HSC of PU.

Code Name	Molar Ratio				HSC (%)
	PCL	PO	HMDI	BDO	
PU0	1	0	6	5	50.31
PU10	0.9	0.1	6	5	53.47
PU20	0.8	0.2	6	5	56.89
PU30	0.7	0.3	6	5	60.62
PU40	0.6	0.4	6	5	64.69

**Table 2:** The DSC parameters of PU.

Sample	T_g_ (°C)	T_m,PO_ (°C)	$∆H_{m}$_,PO_ (J/g)	T_m,PCL_ (°C)	$∆H_{m}$_,PCL_ (J/g)	X_c,PCL_ (%)	T_m,BD_ (°C)	$∆H_{m}$_,BD_ (J/g)
PU0	−55.50	-	-	51.33	0.64	0.91	76.92	5.99
PU10	−53.98	-	-	52.25	0.34	0.51	78.75	5.28
PU20	−57.21	-	-	53.67	0.84	1.28	80.25	6.53
PU30	−62.54	14.33	1.25	44.25	0.24	0.37	74.50	5.50
PU40	−54.00	12.17	0.75	50.92	0.43	0.43	77.83	6.66

**Table 3:** The tensile testing results.

Sample	Young’s Modulus (MPa)	Tensile Strength (MPa)	Elongation at Break (%)
PU0	88.60 ± 3.20 × 10^−2^	10.15 ± 2.09	113.64 ± 15.37
PU10	81.40 ± 6.26 × 10^−2^	15.70 ± 1.22	118.82 ± 10.18
PU20	155.60 ± 6.22 × 10^−2^	13.01 ± 2.15	191.67 ± 22.16
PU30	327.60 ± 2.80 × 10^−2^	22.64 ± 5.54	184.13 ± 12.43
PU40	235.60 ± 0.82 × 10^−2^	20.75 ± 7.02	145.43 ± 22.52

## Data Availability

The original contributions presented in the study are included in the article; further inquiries can be directed to the corresponding author.
